# The immunohistochemical expression of SSTR2A is an independent prognostic factor in meningioma

**DOI:** 10.1007/s10143-021-01651-w

**Published:** 2021-10-02

**Authors:** Christina Fodi, Marco Skardelly, Johann-Martin Hempel, Elgin Hoffmann, Salvador Castaneda, Ghazaleh Tabatabai, Jürgen Honegger, Marcos Tatagiba, Jens Schittenhelm, Felix Behling

**Affiliations:** 1 Department of Neurosurgery, University Hospital Tübingen, Eberhard-Karls-University Tübingen, Hoppe-Seyler Street 3, Tübingen, Germany; 2 Center for CNS Tumors, Comprehensive Cancer Center Tübingen-Stuttgart, University Hospital Tübingen, Eberhard-Karls-University Tübingen, Tübingen, Germany; 3 Department of Diagnostic and Interventional Neuroradiology, University Hospital Tübingen, Eberhard-Karls-University Tübingen, Tübingen, Germany; 4 Department of Radiation-Oncology, University Hospital Tübingen, Eberhard-Karls-University Tübingen, Tübingen, Germany; 5 Department of Nuclear Medicine, University Hospital Tübingen, Eberhard-Karls-University Tübingen, Tübingen, Germany; 6 Department of Neurology & Interdisciplinary Neuro-Oncology, University Hospital Tübingen, Eberhard-Karls-University Tübingen, Tübingen, Germany; 7 Hertie Institute for Clinical Brain Research, Tübingen, Germany; 8 German Cancer Consortium (DKTK), DKFZ Partner Site Tübingen, Tübingen, Germany; 9 Department of Neuropathology, University Hospital Tübingen, Eberhard-Karls-University Tübingen, Tübingen, Germany

**Keywords:** Meningioma, Prognosis, Recurrence-free survival, Somatostatin receptor, SSTR, Immunohistochemistry, Tissue microarray

## Abstract

The expression of somatostatin receptors in meningioma is well established. First, suggestions of a prognostic impact of SSTRs in meningioma have been made. However, the knowledge is based on few investigations in small cohorts. We recently analyzed the expression of all five known SSTRs in a large cohort of over 700 meningiomas and demonstrated significant correlations with WHO tumor grade and other clinical characteristics. We therefore expanded our dataset and additionally collected information about radiographic tumor recurrence and progression as well as clinically relevant factors (gender, age, extent of resection, WHO grade, tumor location, adjuvant radiotherapy, neurofibromatosis type 2, primary/recurrent tumor) for a comprehensive prognostic multivariate analysis (*n* = 666). The immunohistochemical expression scores of SSTR1, 2A, 3, 4, and 5 were scored using an intensity distribution score ranging from 0 to 12. For recurrence-free progression analysis, a cutoff at an intensity distribution score of 6 was used. Univariate analysis demonstrated a higher rate of tumor recurrence for increased expression scores for SSTR2A, SSTR3, and SSTR4 (*p* = 0.0312, *p* = 0.0351, and *p* = 0.0390, respectively), while high expression levels of SSTR1 showed less frequent tumor recurrences (*p* = 0.0012). In the Kaplan–Meier analysis, a higher intensity distribution score showed a favorable prognosis for SSTR1 (*p* = 0.0158) and an unfavorable prognosis for SSTR2A (0.0143). The negative prognostic impact of higher SSTR2A expression remained a significant factor in the multivariate analysis (RR 1.69, *p* = 0.0060). We conclude that the expression of SSTR2A has an independent prognostic value regarding meningioma recurrence.

**Supplementary Information:**

The online version contains supplementary material available at 10.1007/s10143-021-01651-w.

## Introduction

Meningiomas represent the most common benign intracranial tumor [1]. Most cases can be treated effectively by surgical resection, but treatment of recurrent tumors can be challenging. Besides surgery and radiotherapy, no established treatment options for meningiomas exist [2]. Recently, the role of early surgical intervention for recurrent meningioma has been suggested [3]. Therefore, it is important to improve the identification of patients with an increased risk of recurrence. This has also been expressed by the update of the WHO classification of central nervous system tumors in 2016. Since then, invasion of central nervous system tissue by meningioma cells has been established as a standalone criterion for atypia [4]. A DNA methylation–based classification has been proclaimed as superior to the WHO classification for deciding for adjuvant radiation [5]. Molecular findings associated with increased tumor recurrence risk such as TERT promotor mutation and CDKN2A/B deletion are seen only in a small subset of the tumors [6]. Besides these recently described aspects, the detection of patients with an increased risk of recurrence is still insufficient.

One feature of meningiomas that has been utilized for a novel targeted treatment approach is the expression of somatostatin receptors (SSTR2) [7, 8]. Peptide receptor radionuclide therapy seems to be an effective measure for challenging and recurrent meningiomas [9, 10]. Furthermore, a few studies with small meningioma cohorts have focused on the effect of the expression of SSTRs on proliferation and prognosis with mixed results [11–13]. So far, the prognostic potential of SSTR expression in meningiomas has not been further clarified. We have recently described the expression of SSTR1-5 in a large cohort of over 700 meningiomas and characterized expression differences in clinical subgroups such as WHO grade and neurofibromatosis 2 [14]. Due to the importance of more accurate prognostic evaluation of meningiomas, we expanded our initial dataset and collected information about radiographic tumor recurrence and progression as well as other established prognostic factors. The goal of this retrospective study is to evaluate the prognostic role of somatostatin receptor expressions in meningiomas and its impact on tumor recurrence in a large retrospective cohort with a comprehensive prognostic multivariate analysis.

## Materials and methods

### Patient cohort

Data on the immunohistochemical expression of SSTR1-5 in a cohort of 724 meningiomas together with clinical characteristics (gender, age, primary/recurrent tumor, prior radiotherapy, neurofibromatosis type 2, localization, and WHO classification) was collected from our previous study [14]. For the prognostic evaluation, we additionally gathered information about radiographic tumor recurrence/progression as well as applied adjuvant radiotherapy between surgery and tumor recurrence/progression and the extent of resection according to Simpson grade. Tumor recurrence was defined if a clear tumor recurrence was seen and tumor progression if residual tumor showed clear progression on follow-up imaging. All radiographic reports and images were critically reviewed for this study. Complete follow-up data was available for 666 cases. Sufficient staining results were attained for 650, 654, 654, 655, and 657 cases for SSTR1, SSTR2A, SSTR3, SSTR4, and SSTR5, respectively.

### Tissue microarray and immunohistochemistry

Existing tissue microarray (TMA) data of 666 meningiomas that were stained for SSTR 1 (Gramsch, Schwabhausen, Germany), SSTR2 (Dianova, Hamburg, Germany analysis), SSTR3 (Abcam, Cambridge, United Kingdom), SSTR4 (Gentex, Zeeland, USA), and SSTR5 (Abcam, Cambridge, UK) were examined. Two sample cylinders of 1 mm diameter were extracted for each tumor for TMA construction. For TMA construction and immunohistochemistry protocol, details are shown in Behling et al. [14].

### Microscopic assessment and statistical methods

An intensity distribution score described by Barresi et al. for immunohistochemical evaluation of somatostatin receptors was used to summarize expression data [12]. The immunostaining intensity was scored as negative (0), weak (1), intermediate (2), or strong (3). The area of positivity was estimated as follows: 0 (0–5%), 1 (5–25%), 2 (25–50%), 3 (50–75%), and 4 (75–100%). The intensity score was multiplied by the area of positivity, leading to an intensity distribution score of 0–12. The cutoff for dichotomizing the cohort was chosen at a score > / = 6. The Pearson’s chi-squared and log-rank tests were used for univariate and the cox proportional hazard model for multivariate analysis. A significance level of α < 0.05 was applied. A classification and regression tree (CART) analysis was used to define prognostic cutoff for age.

## Results

### Clinical characteristics and SSTR expression of the study cohort

The basic clinical characteristics and the immunohistochemical expression scores of the somatostatin receptors 1, 2A, 3, 4, and 5 of our study cohort have been described in detail in our previous work [14]. We have provided a supplementary table with the distribution and clinical characteristics of this cohort which displays only 666 cases of the original 726 tumors since follow-up data was only available for 91.7% (Supplementary Table 1). The mean follow-up was 27.7 months ranging from 1.1 to 195.6 months. Complete resection was achieved in 70.9% (Simpson grade I, II, and III), and a subtotal resection was done in 29.1% (Simpson grade IV or V). The decision for adjuvant radiation therapy was made in 33 cases (5%). Details are displayed in Table 1. We collected information about radiographic tumor recurrence and progression as well as other established prognostic factors. Tumor recurrence or progression of residual tumor was observed in 137 of 666 cases (20.6%).

**Table 1:** Univariate analysis of prognostic factors in meningioma (Pearson’s chi-squared test)

		Tumor recurrence		
	*N* (%)	Yes	No	*p*-value (Prob > Chisq)
Gender				
Female Male	462 (69.4) 204 (30.6)	67 (14.5) 70 (34.3)	395 (85.5) 134 (65.7)	< 0.0001*
Age				
< 39.48 > = 39.48	91 (13.7) 575 (86.3)	35 (38.5) 102 (17.7)	56 (61.5) 473 (82.3)	< 0.0001*
Recurrent meningioma				
Primary Recurrence	562 (84.4) 104 (15.6)	491 (87.4) 38 (36.5)	71 (12.6) 66 (63.5)	< 0.0001*
Adjuvant radiotherapy				
Yes No	33 (5.0) 633 (95.1)	8 (24.2) 129 (20.4)	25 (75.8) 504 (79.6)	0.5925
Simpson grade				
< 4 > = 4	466 (70.9) 191 (29.1)	64 (13.7) 72 (37.7)	402 (86.3) 119 (62.3)	< 0.0001*
WHO classification 2016				
I	538 (80.8)	64 (11.9)	474 (88.1)	< 0.0001*
II III	106 (15.9) 22 (3.3)	54 (50.9) 19 (86.4)	52 (49.1) 3 (13.6)	
NF2				
Yes No	62 (9.3) 604 (90.7)	27 (43.6) 110 (18.2)	35 (56.5) 494 (81.8)	< 0.0001*
Location				
Convexity/falx Skull base Spinal	265 (39.8) 340 (51.1) 61 (9.2)	63 (23.8) 70 (20.6) 4 (6.6)	202 (76.2) 270 (79.4) 57 (93.4)	0.0111*
SSTR1 (*n* = 651)				
> / = 6.0 < 6.0	415 (63.8) 236 (36.3)	70 (16.9) 65 (27.5)	345 (83.1) 171 (72.5)	0.0012*
SSTR2A (n = 655)				
> / = 6.0 < 6.0	293 (44.7) 362 (55.3)	71 (24.2) 63 (17.4)	222 (75.8) 299 (82.6)	0.0312*
SSTR3 (*n* = 655)				
> / = 6.0 < 6.0	42 (6.4) 613 (93.6)	14 (33.2) 121 (19.7)	28 (66.7) 492 (80.3)	0.0351*
SSTR4 (*n* = 656)				
> / = 6.0 < 6.0	24 (3.7) 632 (96.3)	9 (37.5) 127 (20.1)	15 (62.5) 505 (79.9)	0.0390*
SSTR5 (*n* = 658)				
> / = 6.0 < 6.0	189 (28.7) 469 (71.3)	41 (21.7) 95 (20.3)	148 (78.3) 374 (79.7)	0.6804

*Abbreviations:* *SSTR* somatostatin receptor, *NF2* neurofibromatosis type 2, *WHO* World Health Organization; the asterisk (*) presents statistically significant results

### Univariate analysis

The results of the univariate analysis using the intensity distribution score cutoff at > / = 6 are displayed in Table 1. For SSTR1, 415 cases (63.8%) reached a score of 6 or higher, while 236 (36.3%) scored below the cutoff. Tumors with a lower expression score had a higher recurrence rate (27.5% vs. 16.9%, *p* = 0.0012). An intensity distribution score of 6 or higher for SSTR2A was observed in 293 tumor (44.7%) and below in 362 (55.3%) cases. Higher expression was associated with an increased recurrence rate (24.2% compared to 17.4%, *p* = 0.0312). The expression scores for SSTR3 and SSTR4 were quite low. For SSTR3, only 42 cases reached an intensity distribution score of 6 or higher (6.4%), and with 93.6% (*n* = 613), the majority of tumors scored below the cutoff. An even lower number of higher expression scores (> / = 6) was observed with 24 cases (3.7%). For both, SSTR3 and SSTR4, the few cases with higher expressions had a higher rate of tumor recurrences with 33.2% (*p* = 0.0351) and 37.5% (*p* = 0.0390), respectively. An intensity distribution score above the cutoff of 6 was observed in 189 cases for SSTR5 (28.7%). There was no significant difference in the rate of tumor recurrence compared to the 469 tumors with a lower expression score.

**Fig. 1:**
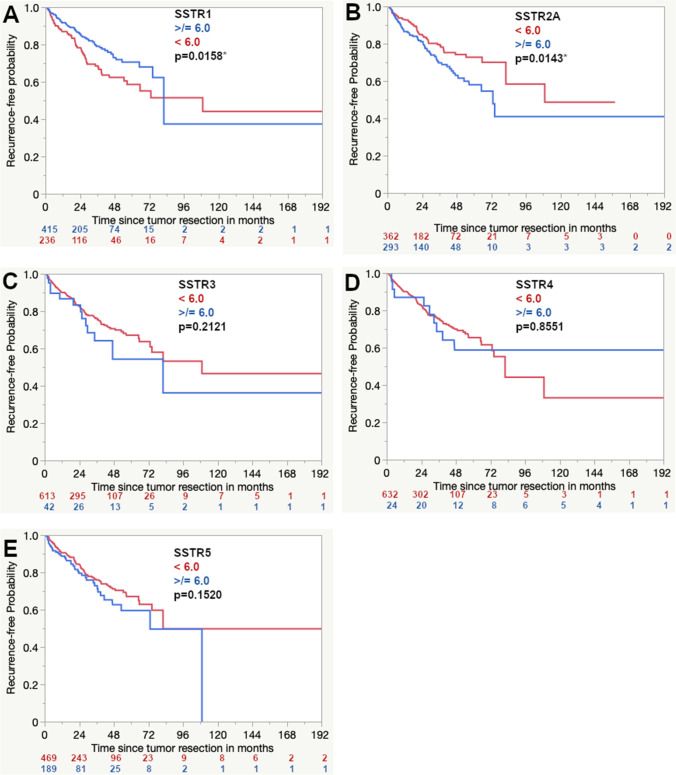
Univariate analysis of the prognostic impact of the expression of SSTR1 (**A**), SSTR2A (**B**), SSTR3 (**C**), SSTR4 (**D**), and SSTR5 (**E**). The asterisks (*) represent statistically significant differences

**Fig. 2:**
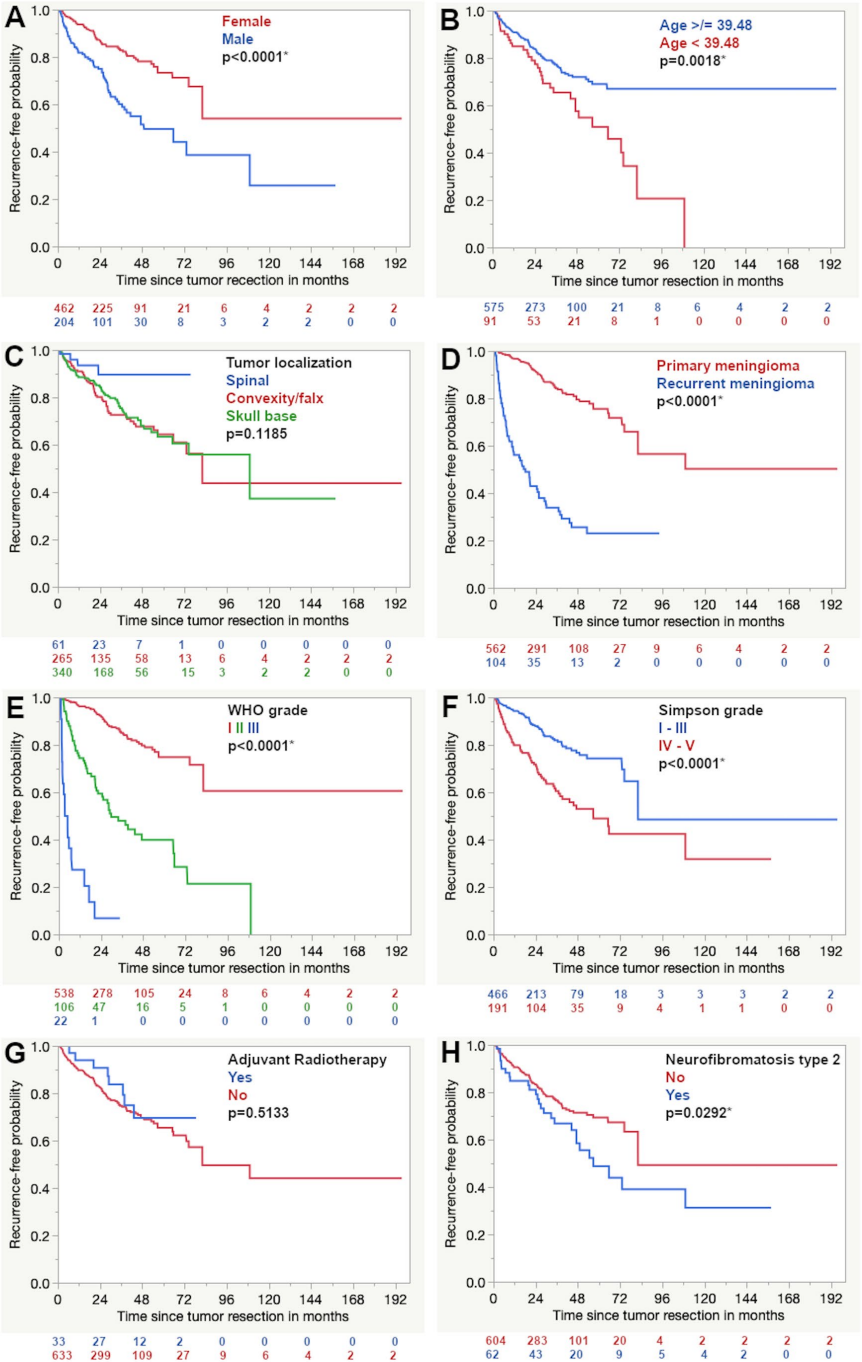
Univariate analysis of the prognostic impact of the expression of gender (**A**), age (**B**), tumor localization (**C**), recurrent tumor (**D**), WHO grade (**E**), extent of resection according to Simpson (**F**), adjuvant radiotherapy (**G**), and neurofibromatosis type 2 (**H**). The age cutoff is set according to a CART analysis. The asterisks (*) represent statistically significant differences

In the Kaplan–Meier analysis, higher expression of SSTR1 and lower expression scores of SSTR2A showed a more favorable progression-free survival (Fig. 1). The univariate results of all other included prognostic factors are shown in Table 1 and Fig. 2.

### Multivariate analysis

All clinical factors were included in the multivariate analysis together with the intensity distribution scores for SSTR1-5. Male gender, younger age, recurrent tumor, incomplete resection, and higher WHO grade were risk factors for tumor recurrence (*p* = 0.0042, *p* = 0.0380, *p* < 0.0001, *p* = 0.0005, and *p* < 0.0001, respectively). Adjuvant radiotherapy was an independent positive prognostic factor (*p* = 0.0004). Neurofibromatosis type 2 and tumor location lost its prognostic significance when the other predictors of recurrence were included in a multivariate analysis (compared to the univariate analysis, see Table 1).

Higher intensity distribution scores for SSTR2A remained an independent negative prognostic factor (*p* = 0.0060), while SSTR1, SSTR3, SSTR4, and SSTR5 had no influence on progression-free survival. The details of the multivariate analysis are displayed in Table 2, and corresponding graphs are shown in Fig. 1.

**Table 2:** Multivariate analysis including immunohistochemical expression of SSTR1-5 (Cox proportional hazard)

	Risk ratio (95%CI)	*p*-value (Prob > Chisq)
Male gender	1.77 (1.20–2.63)	0.0042*
Age < 39.48 Recurrent meningioma NF2	1.84 (1.03–3.27) 4.74 (3.16–7.10) 0.64 (0.32–1.27)	0.0380* < 0.0001* 0.2029
Location		
Convexity/falx vs. skull base Convexity/falx vs. spinal Skull base vs. spinal	0.84 (0.57–1.25) 1.27 (0.44–3.64) 1.50 (0.53–4.25)	0.3943 0.6612 0.4464
Adjuvant radiotherapy Simpson grade > / = 4	0.25 (0.12–0.54) 1.97 (1.35–2.88)	0.0004* 0.0005*
WHO classification 2016		
I vs. II I vs. III II vs. III SSTR1 > / = 6 SSTR2A > / = 6 SSTR3 > / = 6 SSTR4 > / = 6 SSTR5 > / = 6	0.29 (0.19–0.44) 0.06 (0.03–0.11) 0.20 (0.11–0.39) 0.86 (0.59–1.26) 1.69 (1.16–2.46) 0.77 (0.39–1.52) 0.65 (0.29–1.47) 1.20 (0.81–1.80)	< 0.0001* < 0.0001* < 0.0001* 0.4327 0.0060* 0.4497 0.3029 0.3687

*Abbreviations:* *SSTR* somatostatin receptor, *NF2* neurofibromatosis type 2, *WHO* World Health Organization; the asterisk (*) presents statistically significant results

## Discussion

The improvement of the prognostic assessment of meningiomas beyond the current WHO classification has been investigated for many years and led to the update of the WHO classification for meningiomas in 2016 with CNS invasion being now regarded as a stand-alone criterion for atypia [4]. Additionally, a DNA methylation–based classification and TERT promoter mutation status analysis are among the most promising advances with the potential to further refine the prognostic evaluation of meningiomas [5, 15] but currently do not provide a target for treatment.

The expression of somatostatin receptors in meningiomas has been utilized for the implementation of peptide radio receptor therapy in selected cases with reports of good efficacy [9, 10]. We have recently described the distribution of the somatostatin receptors 1, 2A, 3, 4, and 5 in a large meningioma cohort using immunohistochemical staining and a semiquantitative assessment score to help form the basis for the development of a differentiated and improved peptide radioreceptor therapy [14]. We observed differences in the SSTR expression in certain clinical and histopathological subgroups. Among these subgroups, a lower expression of SSTR1 was seen in recurrent tumors compared to primary meningiomas, while no differences regarding other SSTRs were observed. Now we present the first multivariate evaluation of all somatostatin receptors and its prognostic impact on meningioma recurrence in a comprehensive cohort of 666 meningiomas. We combined qualitative and quantitative data from immunohistochemistry into an immunoreactive score and added clinical prognostic factors to a multivariate analysis. We show that high SSTR2A expression is an independent predictor for unfavorable progression-free survival in meningioma. Possibly a main factor for this result is the observation of a higher rate of SSTR2A expression for grade II meningiomas compared to grade I tumors, which we published recently. Interestingly, the expression rate for grad III tumors is lower, which may express the dedifferentiation of malignant meningiomas [14]. Downregulation of multiple previously strongly expressed genes is frequently observed in recurring meningiomas [16].

Somatostatin receptors are G-protein-coupled transmembrane receptors that regulate numerous functions in different organs [17]. All five SSTRs seem to act by inhibition of the adenylate cyclase and subsequent stimulation of phospholipase C and calcium mobilization [18, 19]. Multiple downstream pathways of somatostatin have been identified resulting in several antiproliferative mechanisms in different tumors, mainly involving the inhibition of cell cycle progression, apoptosis, and angiogenesis [19, 20]. However, high expression levels have been demonstrated in different tumor types, including neuroendocrine tumors, renal cell carcinomas, lymphomas, and meningiomas [19]. If SSTR expression is functionally linked to tumor formation or proliferation, in contrary the known function of SSTRs is unclear. It is important to emphasize that the current knowledge is based on studies in different tumor types and models.

The application of somatostatin showed a dramatic decrease of the incidence of bladder cancer due to the carcinogenic effect of nitrosamine in rats, suggesting a protective effect possibly by supporting apoptosis of dysplastic cells at risk malignification [21]. One possible explanation is that the expression of SSTRs is a last signal response by hormonal proapoptotic influence of cells before further dedifferentiation and tumor formation. Therefore, increased SSTR expression may be a byproduct of increased proliferation and may not necessarily be actively involved in tumor growth.

On the contrary, an older in vitro study revealed that the application of somatostatin and octreotide resulted in increased cell growth of a meningioma cell line, suggesting a stimulatory proliferative effect in meningioma [22]. However, the mechanism of how differences in SSTR expressions may reflect tumor progression remains unclear.

There is some data on the prognostic role of SSTR expression in smaller meningioma series. In 2004, Arena et al. analyzed the expression of SSTR1-5 mRNA in 42 meningiomas and found no correlation with the immunohistochemical expression of MIB-1 and bcl-2, markers for proliferation and apoptosis, respectively. Additionally, a decrease in DNA synthesis was observed when somatostatin was applied to primary cell cultures derived from meningiomas [11]. On the contrary, Barresi et al. evaluated the immunohistochemical expression of SSTR2A in 35 meningiomas. A higher rate of immunopositivity was seen in higher grade meningiomas, and a correlation with increased micro-vessel density and MIB-1 expression was demonstrated [12], suggesting a possible link between more aggressive meningiomas and higher SSTR2A expression. Now our study confirms that a higher expression of SSTR2A is an independent predictor for increased risk meningioma recurrence.

In 2015, a similar but contrary context was described for SSTR5, in the largest study on SSTR expression in meningiomas so far by Silva and colleagues. Sixty meningiomas were assessed regarding all 5 somatostatin receptors. SSTR5 was more frequently observed in grade I meningiomas. However, a correlation of SSTR5 expression with tumor recurrence or regrowth was not seen [13]. This may have been based on the small number of included cases. However, Silva et al. first described a possible connection of SSTR5 expression and more benign meningioma types. This is in line with the results of our previous study, where we were able to show that the expression of SSTR5 is lower in WHO grade III meningiomas [14]. However, the results of our study do not show a prognostic impact of SSTR5 in the univariate and multivariate analysis.

In summary, previous suggestions of a possible prognostic impact of SSTR expressions in meningioma have now been confirmed in a large retrospective analysis with the inclusion of established prognostic factors in a comprehensive multivariate analysis. Incorporation of SSTR2A into the routine neuropathology staining panel of meningioma may provide additional information regarding tumor progression in meningioma and help in the decision to indicate radiotherapy in otherwise prognostically uncertain cases. However, the mechanism behind the contrary independent prognostic impact of the expression of SSTR2A remains a matter for further research.

The main limitation of this study is its retrospective design and variable follow-up. Longer follow-up periods are desirable. Additionally, including molecular factors that are associated with tumor recurrence/progression would be of interest, such as the *TERT* promoter mutation [15]. Furthermore, the amount of meningioma tissue for TMA construction and immunohistochemistry may not be adequate in case of heterogenous staining. However, two biopsy cylinders with a diameter of 1 mm each were extracted and analyzed for each tumor, which is a sufficient amount to minimize heterogeneity in meningioma. We chose to utilize the intensity distribution score that was described by Barresi et al. in 2008 [12] because it incorporates the staining intensity and quantification. Still the scoring system has certain limitations. A score of 6, for example, can express maximum staining intensity (intensity score of 3) in 25–50% of tumor cells (quantification score 2) or a mediocre staining intensity (score 2) in 50–75% of tumor cells (quantification score 3). Both tumors received an intensity distribution score of 6. It is debatable if tumors with such differences in staining should be graded the same. Further insight into the quantification in comparison with different scoring methods in immunohistochemistry and prognostic data would be of great help to improve the scoring system.

## Conclusion

An increased expression of SSTR2A is an independent negative prognostic factor for meningioma recurrence.

## Supplementary Information

Below is the link to the electronic supplementary material.

Supplementary file1 (DOC 55 KB)

## Data Availability

The dataset is available upon reasonable request.
