# Correction to: The immunohistochemical expression of SSTR2A is an independent prognostic factor in meningioma

**DOI:** 10.1007/s10143-022-01817-0

**Published:** 2022-05-23

**Authors:** Christina Fodi, Marco Skardelly, Johann-Martin Hempel, Elgin Hoffmann, Salvador Castaneda, Ghazaleh Tabatabai, Jürgen Honegger, Marcos Tatagiba, Jens Schittenhelm, Felix Behling

**Affiliations:** 1 Department of Neurosurgery, University Hospital Tübingen, Eberhard-Karls-University Tübingen, Hoppe-Seyler Street 3, Tübingen, Germany; 2 Center for CNS Tumors, Comprehensive Cancer Center Tübingen-Stuttgart, University Hospital Tübingen, Eberhard-Karls-University Tübingen, Tübingen, Germany; 3 Department of Diagnostic and Interventional Neuroradiology, University Hospital Tübingen, Eberhard-Karls-University Tübingen, Tübingen, Germany; 4 Department of Radiation-Oncology, University Hospital Tübingen, Eberhard-Karls-University Tübingen, Tübingen, Germany; 5 Department of Nuclear Medicine, University Hospital Tübingen, Eberhard-Karls-University Tübingen, Tübingen, Germany; 6 Department of Neurology & Interdisciplinary Neuro-Oncology, University Hospital Tübingen, Eberhard-Karls-University Tübingen, Tübingen, Germany; 7 Hertie Institute for Clinical Brain Research, Tübingen, Germany; 8 German Cancer Consortium (DKTK), DKFZ Partner Site Tübingen, Tübingen, Germany; 9 Department of Neuropathology, University HospitalTübingen, Eberhard-Karls-University Tübingen, Tübingen, Germany

**Correction to: Neurosurgical Review**

10.1007/s10143-021-01651-w

The authors regret that the version of Figure 1 that appears in the original article is incorrect.

The original article has been corrected.

