# Supplementary material for: The immunohistochemical expression of SSTR2A is an independent prognostic factor in meningioma
Source: Neurosurg Rev. 2021 Oct 2;45(4):2671–9. doi: 10.1007/s10143-021-01651-w (PMC9349155; doi:10.1007/s10143-021-01651-w)
Supplement: Supplementary file 1 — Supplementary file1 (DOC 55 KB) [file 10143_2021_1651_MOESM1_ESM.doc]

Supplementary Table1: Cohort characteristics and immunohistochemical distribution of somatostatin receptor expression (ANOVA).

|  |  | **SSTR 1 (n=650)** | | **SSTR2A (n=654)** | | **SSTR 3 (n=654)** | | **SSTR 4 (n=655)** | | **SSTR 5 (n=657)** | |
| --- | --- | --- | --- | --- | --- | --- | --- | --- | --- | --- | --- |
|  | **N (%)** | **Mean (95%CI)** | **p-value** | **Mean (95%CI)** | **p-value** | **Mean (95%CI)** | **p-value** | **Mean (95%CI)** | **p-value** | **Mean (95%CI)** | **p-value** |
| **Gender**  Female  Male    **Age**  >=39.48  <39.48  **Recurrence**  Primary  Recurrence  **Prior radiation**  Yes  No  **NF2**  Yes  No  **Localization**  Convexity/Falx  Skull base  Spinal  **2016 WHO classification**  I  II  III  **Tumor recurrence**  Yes  No | 462 (69.4)  204 (30.6)  575 (86.3)  91 (13.7)  562 (84.4)  104 (15.6)  58 (8.7)  608 (91.3)  62 (9.3)  604 (90.7)  265 (39.8)  340 (51.1)  61 (9.2)  538 (80.8)  106 (15.9)  22 (3.3)  137 (20.6)  529 (79.4) | 6.7 (6.5-6.9)  6.2 (5.9-6.6)  6.7 (6.5-6.9)  5.7 (5.2-6.3)  6.8 (6.5-7.0)  5.5 (5.0-6.0)  5.5 (4.8-6.2)  6.7 (6.5-6.9)  4.8 (4.2-5.5)  6.7 (6.5-7.0)    5.8 (5.5-6.2)  6.9 (6.6-7.1)  8.1 (7.4-8.8)  6.8 (6.6-7.0)  5.7 (5.2-6.2)  4.9 (3.7-6.0)  5.5 (5.0-5.9)  6.9 (6.6-7.1) | 0.0429*  0.0022*  <.0001*  0.0023*  <.0001*  <.0001*  <.0001*  <.0001* | 5.8 (5.6-6.1)  6.0 (5.7-6.4)  6.0 (5.8-6.2)  5.5 (5.0-6.0)  5.8 (5.6-6.1)  6.2 (5.7-6.7)  6.1 (5.4-6.8)  5.9 (5.7-6.1)  5.3 (4.6-6.0)  6.0 (5.8-6.2)  5.6 (5.3-6.0)  6.3 (6.0-6.5)  5.0 (4.3-5.6)  5.8 (5.6-6.0)  6.5 (6.1-7.0)  5.4 (4.3-6.4)  6.2 (5.8-6.6)  5.8 (5.6-6.0) | 0.4447  0.1128  0.1944  0.5608  0.0566  0.0002*  0.0158*  0.1315 | 2.2 (2.0-2.4)  2.3 (2.0-2.6)  2.1 (1.9-2.3)  2.8 (2.4-3.3)  2.2 (2.0-2.4)  2.3 (1.9-2.8)  2.1 (1.5-2.6)  2.2 (2.1-2.4)  3.7 (3.2-4.3)  2.1 (1.9-2.2)  1.7 (1.5-2.0)  2.7 (2.5-2.9)  1.7 (1.1-2.2)  2.2 (2.1-2.4)  2.1 (1.7-2.5)  2.0 (1.1-2.9)  2.4 (2.0-2.8)  2.2 (2.0-2.4) | 0.3859  0.0041*  0.5015  0.6519  <.0001*  <.0001*  0.7100  0.2674 | 2.8 (2.6-2.9)  2.7 (2.4-2.9)  2.7 (2.5-2.8)  3.1 (2.8-3.4)  2.8 (2.6-2.9)  2.6 (2.3-2.9)  2.5 (2.1-2.9)  2.8 (2.6-2.9)  3.6 (3.2-4.0)  2.7 (2.5-2.8)  2.6 (2.4-2.8)  2.8 (2.6-3.0)  3.2 (2.7-3.6)  2.8 (2.7-3.0)  2.5 (2.2-2.8)  2.1 (1.4-2.7)  2.6 (2.3-2.9)  2.8 (2.6-2.9) | 0.3736  0.0272*  0.3955  0.2456  <.0001*  0.0255*  0.0183*  0.2880 | 4.9 (4.7-5.0)  4.9 (4.6-5.1)  4.9 (4.8-5.1)  4.6 (4.2-4.9)  4.9 (4.7-5.1)  4.7 (4.3-5.0)  4.3 (3.8-4.8)  4.9 (4.8-5.1)  4.2 (3.7-4.6)  4.9 (4.8-5.1)  4.5 (4.3-4.8)  5.0 (4.8-5.1)  5.8 (5.3-6.2)  4.9 (4.8-5.1)  4.8 (4.4-5.1)  3.4 (2.7-4.2)  4.4 (4.1-4.8)  5.0 (4.8-5.1) | 0.9395  0.0461*  0.2440  0.0229*  0.0017*  <.0001*  0.0010*  0.0029* |

Abbreviations: SSTR: Somatostatin Receptor, CI: Confidence Interval, CART: Classification and Regression Tree, NF2: Neurofibromatosis Type 2, WHO: World Health Organization, ANOVA: Analysis of Variance, asterisk(*) presents statistically significant results.
